# A 1 kV Compensation-Free Three-Stage Inductive Voltage Divider with Integrated-Reference Self-Calibration

**DOI:** 10.3390/s26165136

**Published:** 2026-08-14

**Authors:** Jian Wang, Xiaodong Yin, Hao Liu, Junjie Liu, Jian Liu, Shuhui Yi, Jiahao Sun, Junjun Huang, Wenbo Sun

**Affiliations:** 1 China Electric Power Research Institute, Wuhan 430074, China; yinxiaodong@epri.sgcc.com.cn (X.Y.); liuhao1@epri.sgcc.com.cn (H.L.); liujunjie1@epri.sgcc.com.cn (J.L.); liujian@epri.sgcc.com.cn (J.L.); yishuhui@epri.sgcc.com.cn (S.Y.); sunjiahao@epri.sgcc.com.cn (J.S.); hjj_wd@whu.edu.cn (J.H.); sunwenbo@epri.sgcc.com.cn (W.S.); 2 National Center for High Voltage Measurement, Wuhan 430074, China; 3 Key Laboratory of Measurement and Test of High Voltage and Heavy Current, State Administration for Market Regulation, Wuhan 430074, China

**Keywords:** inductive voltage divider, voltage–ratio standard, bootstrap self–calibration, magnetic shielding, harmonic measurement

## Abstract

Electronic compensation networks used in high–accuracy inductive voltage dividers (IVDs) can introduce temperature– and aging–dependent drift sources, complicating long–term uncertainty evaluation. This paper presents a 1 kV, 10–tap, compensation–free three–stage IVD that combines multi–stage excitation, a closed shielded core, and equipotential coaxial–cable windings to suppress excitation, magnetic–coupling, and capacitive errors. An integrated reference winding is embedded in the third–stage coaxial–cable winding, enabling a simplified bootstrap self–calibration procedure without an additional auxiliary IVD. At 50 Hz, the calibrated ratio error and phase displacement are within 0.08 μV/V and 0.08 μrad, respectively, for all taps, with expanded uncertainties of 0.04 μV/V and 0.06 μrad. Wideband measurements up to 3 kHz further show that the proposed IVD can serve as a voltage–ratio reference for harmonic voltage measurement and calibration.

## 1. Introduction

Inductive voltage dividers (IVDs) are widely used as precision AC voltage–ratio standards because their output ratio is determined primarily by winding turns rather than by the absolute values of resistive or capacitive components. This operating principle provides high input impedance, low output impedance, and excellent ratio stability, making IVDs important reference instruments for voltage–ratio traceability, transformer calibration, and precision AC measurement [1,2,3,4,5]. In contrast to resistive dividers, IVDs are less sensitive to long–term changes in material parameters, although residual excitation, capacitive leakage, and magnetic–coupling errors must still be carefully controlled in high–accuracy applications.

Foundational studies in the 1960s and 1970s established IVDs as low–voltage AC ratio standards for precision impedance and voltage–ratio measurements, with an emphasis on self–calibration techniques and frequency extension [6,7,8]. The requirements considered here are different: the proposed divider operates at 1 kV and is intended for voltage–ratio traceability and harmonic voltage–ratio reference applications. At this voltage level, insulation coordination, electromagnetic shielding, compensation–free ratio reproduction, and practical self–calibration become central design considerations.

The common–winding configuration of an IVD also enables self–calibration, which is an important advantage for establishing voltage–ratio traceability [9,10]. Based on frequency division compensation, the National Center for High Voltage Measurement (NCHVM) of China developed a 1000 V IVD achieving a ratio error better than 2 × 10^−7^ over the frequency range of 50 Hz to 1000 Hz [11]. The National Institute of Metrology (NIM) of China developed a 1000 V multi–decade IVD at power frequency with a ratio error better than 5 × 10^−8^ [12,13]. The National Measurement Institute of Australia (NMIA) developed a 1000 V multi–decade IVD operating from 40 Hz to 1000 Hz with output ratios of 0.001 to 0.01, exhibiting a wideband error within 1 × 10^−6^ [14,15].

Beyond the standard 1 kV level, considerable research has been devoted to expanding the voltage and frequency ranges of IVDs. For instance, a 10 kV IVD was developed to bridge the gap between low–voltage standards and high–voltage transformers [16], and a precision three–stage VT with a double–excitation structure was designed to support traceability at the 500 kV level [17]. To address high–frequency performance, a two–stage coaxial design was proposed to minimize capacitive errors at frequencies up to 100 kHz [18]. However, high–accuracy 1 kV IVDs often rely on compensation networks containing resistive, capacitive, or adjustable components [11,12,13]. These components can introduce temperature– and aging–dependent drift mechanisms that are difficult to separate from the intrinsic ratio error of the divider. Eliminating electronic compensation components is therefore attractive for reducing drift–sensitive elements in the ratio–reproduction path and for simplifying uncertainty evaluation.

Calibration methods have evolved in parallel with divider structures. Complex calibration systems have been developed for inductive and resistive dividers over extended frequency ranges [19], and transient approaches based on damped oscillatory waves have been used for rapid frequency characterization of voltage transformers [20]. Harmonic voltage–ratio test systems have also been established to support power–quality measurements [21]. For IVD self–calibration, bifilar–winding techniques [22] and improved bootstrap algorithms [23] have been proposed to reduce reliance on external standards and cumulative error propagation.

Despite these advances, practical implementation remains challenging. Conventional bootstrap calibration generally requires precision auxiliary devices, such as an auxiliary IVD and a guard IVD, to provide stable reference potentials and suppress shielding leakage [23]. In high–voltage and wideband applications, stray electromagnetic parameters, insulation leakage, and shielding configuration further increase system complexity [16,17,18]. These requirements motivate a self–calibration strategy that reduces auxiliary equipment while preserving traceability and ratio accuracy.

To better clarify the relationship between the present work and previous studies, representative IVDs reported in the literature are compared with the proposed IVD in Table 1. The comparison focuses on voltage level, frequency range, calibration approach, reported accuracy, and compensation method.

As shown in Table 1, existing 1 kV IVDs provide excellent power–frequency or moderate wideband accuracy, but this performance is often achieved with compensation networks or relatively complex calibration arrangements. High–voltage and high–frequency extensions further increase the requirements on insulation, shielding, and stray–parameter control. For harmonic voltage–ratio standards, a divider must maintain low ratio error and phase displacement beyond power frequency while remaining traceable and practical to calibrate. These requirements motivate the development of a compensation–free three–stage IVD with an integrated–reference self–calibration method.

This work reports a 1 kV, 10–tap, compensation–free three–stage IVD and an integrated–reference bootstrap self–calibration method. The main contributions are threefold. First, a coaxially nested three–stage structure with a closed shielded core is developed to reduce excitation and magnetic–coupling errors. Second, a multi–wire parallel coaxial–cable winding provides equipotential shielding and suppresses capacitive leakage in the ratio winding. Third, an additional coaxial–cable strand is used as an integrated reference winding, enabling bootstrap self–calibration without a separate auxiliary IVD. The divider is experimentally evaluated at 50 Hz and at frequencies up to 3 kHz.

The remainder of this paper is organized as follows. Section 2 introduces the operating principle of the three–stage IVD. Section 3 describes the structural design and fabrication of the proposed IVD. Section 4 evaluates the effects of magnetic and electrical shielding. Section 5 presents the proposed self–calibration method based on the integrated reference winding. Section 6 reports the calibration results and uncertainty evaluation at 50 Hz. Section 7 discusses the wideband error performance of the IVD, and Section 8 concludes this paper.

## 2. Principles

**Figure 1:**
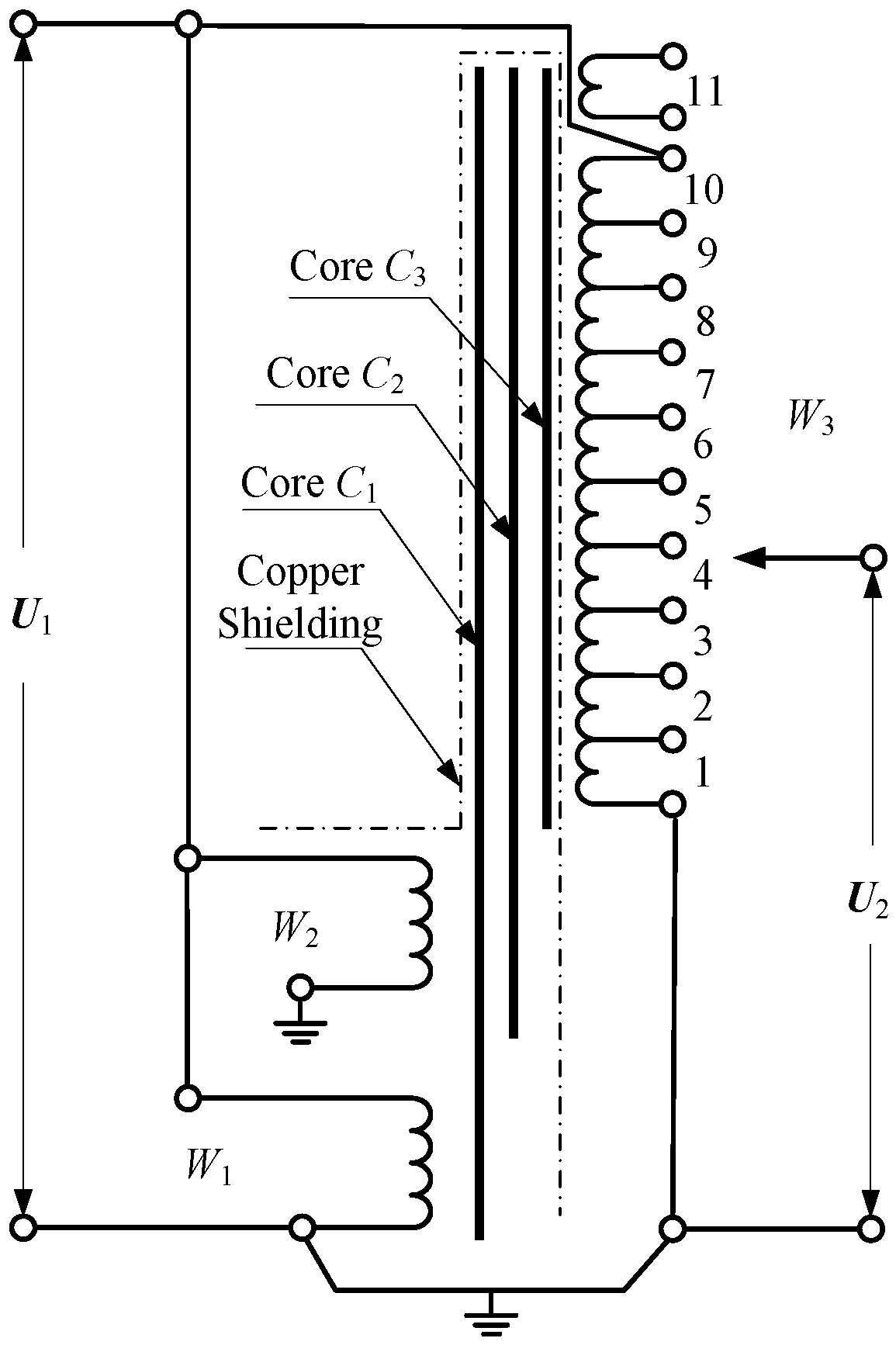
Schematic diagram of the three–stage IVD.

The proposed IVD consists of three cascaded excitation stages, as illustrated in Figure 1. Windings *W*_1_, *W*_2_ and *W*_3_ excite cores *C*_1_, *C*_2_ and *C*_3_, respectively. This multi–stage excitation structure reduces the residual excitation error by successively lowering the excitation current transferred to the ratio winding [24,25,26,27].

Windings *W*_1_ and *W*_2_ each consist of 1000 turns, while *W*_3_ has 1100 turns. *W*_3_ is designed on the autotransformer principle, employing an 11–wire parallel winding technique in which each wire comprises 100 turns. Ten of these wires are connected in series to form the ratio winding, providing voltage ratios of 10:*k* (*k* = 1, 2, …, 10). The remaining winding provides the reference potential for self–calibration, as described in Section 5.

**Figure 2:**
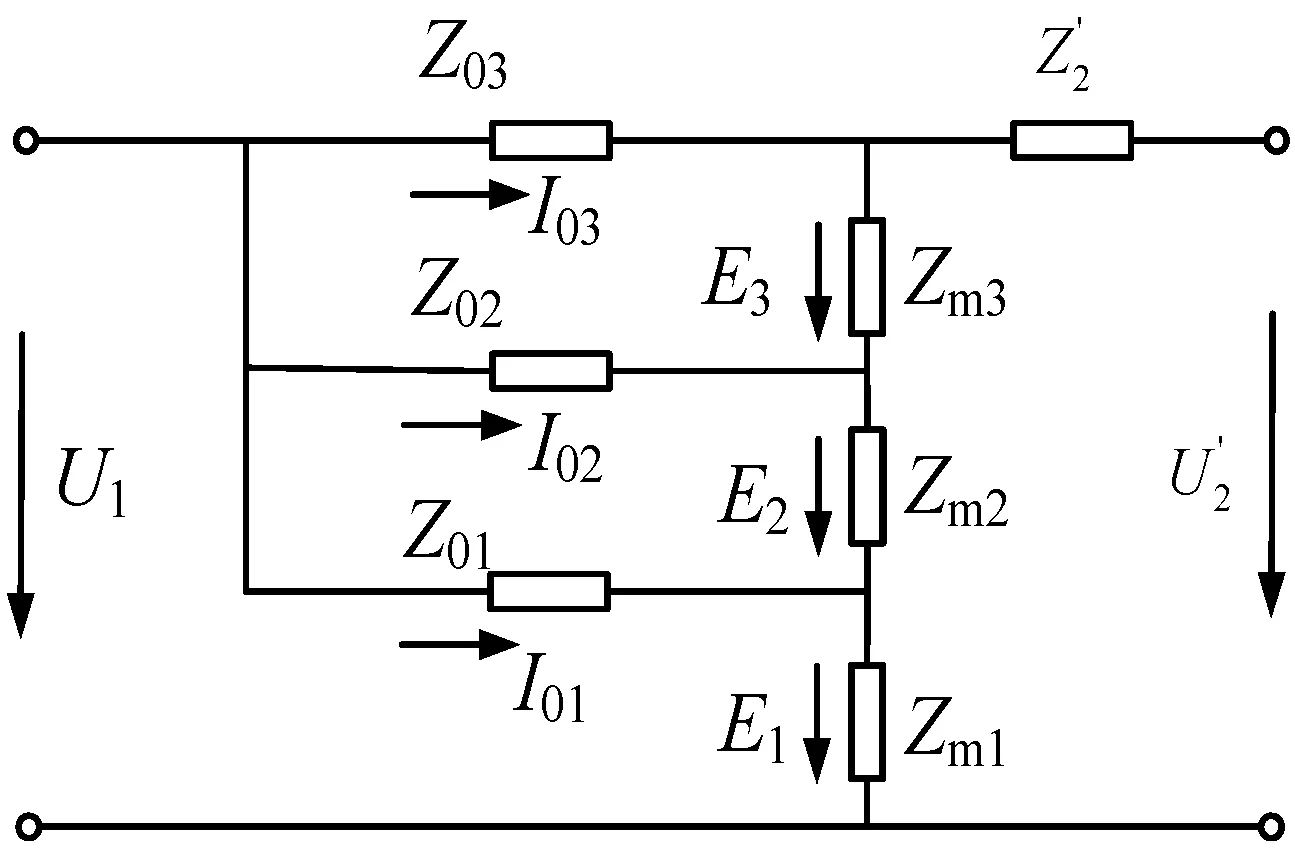
Equivalent circuit of the three–stage IVD.

The equivalent circuit of the IVD, referred to as the primary side, is shown in Figure 2. Here, *I*_01_, *I*_02_ and *I*_03_ represent the excitation currents of the first–, second–, and third–stage VTs, respectively. *Z*_01_, *Z*_02_ and *Z*_03_ denote the impedances, including winding resistance and leakage inductance, of *W*_1,_ *W*_2_ and *W*_3_, respectively. *Z*_m1_, *Z*_m2_ and *Z*_m3_ are the excitation impedances of the first–, second–, and third–stage VTs, respectively. *E*_1_, *E*_2_ and *E*_3_ represent the corresponding electromotive forces.

Under open–circuit (no–load) conditions at the IVD secondary, the excitation error due to the excitation current is given by:

(1) $$\begin{array}{ll} \varepsilon_{e3} & =-\frac{I_{03}Z_{03}}{U_{1}}\\ & =-\frac{I_{01}Z_{01}}{U_{1}}\frac{I_{02}Z_{02}}{I_{01}Z_{01}}\frac{I_{03}Z_{03}}{I_{02}Z_{02}}\\ & =-\varepsilon_{1}\varepsilon_{2}\varepsilon_{3} \end{array}$$

where $\varepsilon_{1}$, $\varepsilon_{2}$ and $\varepsilon_{3}$ are the excitation errors of the first–, second–, and third–stage VTs, respectively. The total excitation error of the IVD equals the negative product of the individual stage excitation errors. Since an excitation error better than 10^−3^ is achievable for each VT stage, the total excitation error of the IVD can theoretically reach 10^−9^ [12,15,17]. For IVDs with errors in the range of 10^−6^ to 10^−5^, error components arising from stray electromagnetic fields cannot be neglected. In such cases, the IVD error is expressed as:

(2) $${\varepsilon \;\prime \;=-\varepsilon }_{1}\varepsilon_{2}\varepsilon_{3}{+\varepsilon }_{c}{+\varepsilon }_{m}$$

Leakage current arising from distributed capacitance gives rise to capacitive error $\varepsilon_{c}$. This distributed capacitance includes turn–to–turn, layer–to–layer, and winding–to–ground components. The magnetic error $\varepsilon_{m}$ arises from non–ideal magnetic coupling between the windings. Nonuniformities in the core and windings cause variations in the induced voltage across each winding section. Consequently, ε ′ is a nonlinear function of the input voltage. Therefore, shielding measures must be implemented during the design and fabrication of the IVD to minimize $\varepsilon_{c}$ and $\varepsilon_{m}$.

## 3. Structural Design and Fabrication

**Figure 3:**
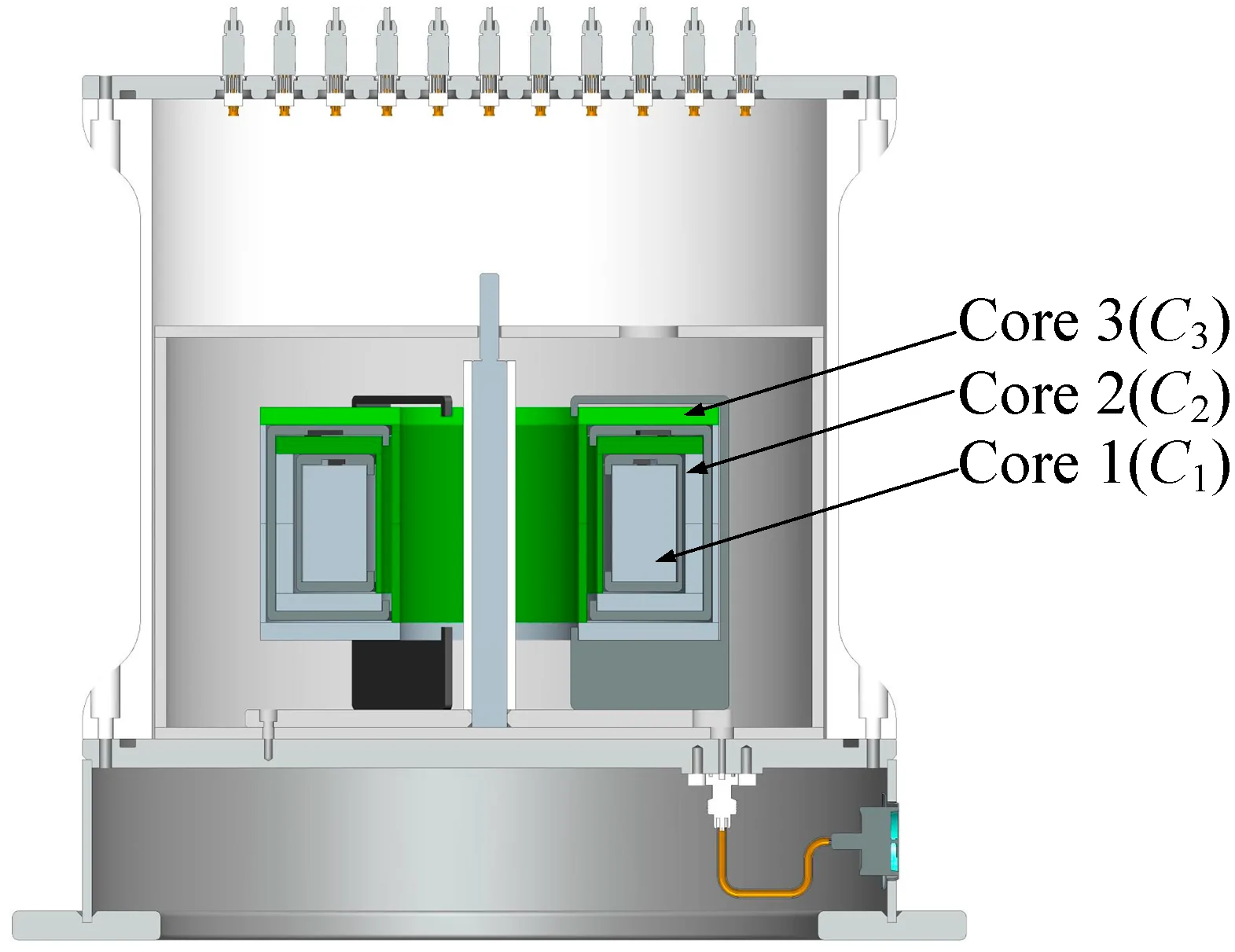
Three–dimensional structure of the three–stage IVD.

**Figure 4:**
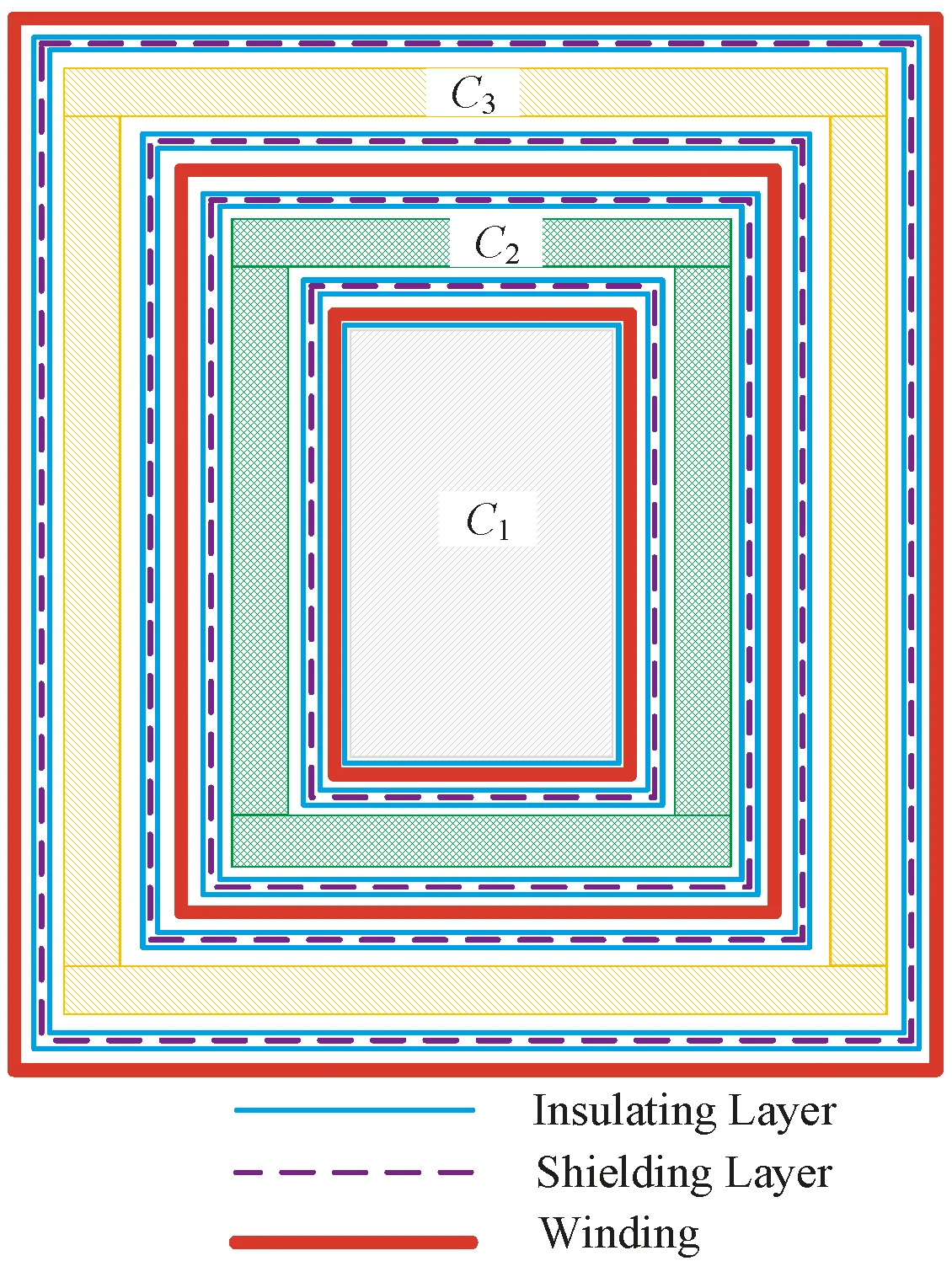
Cross–sectional view of the three–stage IVD.

The physical implementation of the proposed IVD follows a coaxially nested three–stage architecture. As shown in Figure 3 and Figure 4, the first–stage core *C*_1_ and winding *W*_1_ are placed at the innermost position, the second–stage core *C*_2_ surrounds the first–stage assembly and functions as a closed magnetic shield, and the third–stage core *C*_3_ encloses the second–stage assembly. The third–stage coaxial–cable winding *W*_3_ is wound around *C*_3_ to form the ratio winding.

**Figure 5:**
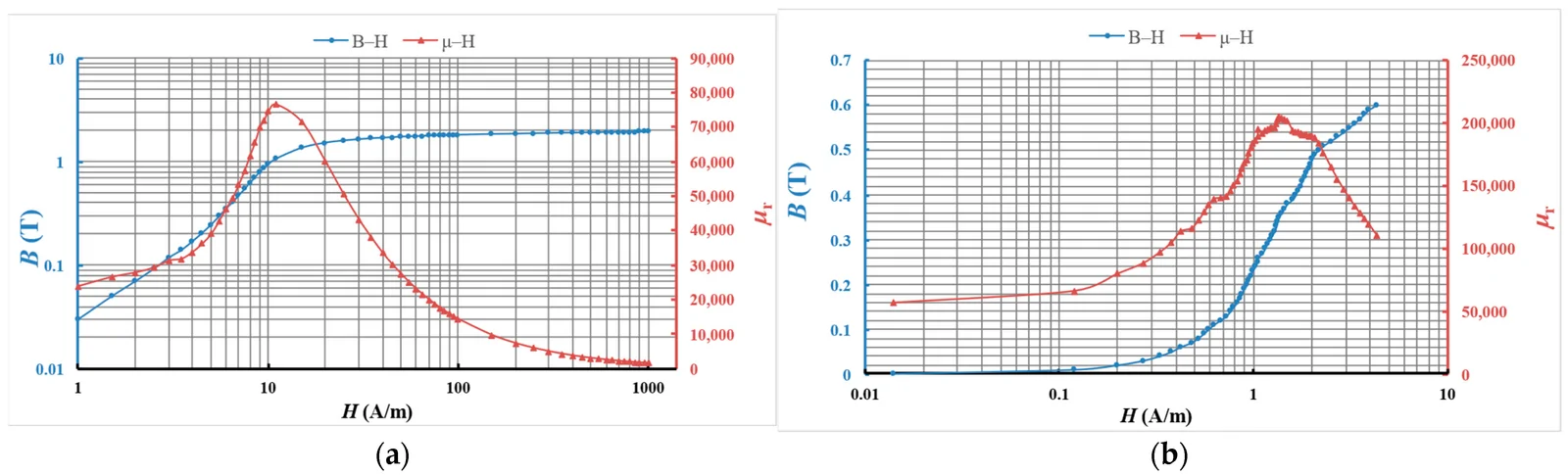
B–H and μ–H curves of core materials: (**a**) cold–rolled silicon steel; (**b**) permalloy.

For the first–stage VT, the excitation winding *W*_1_ consists of 1000 turns of enameled copper wire, uniformly wound around the annular core *C*_1_ in two layers of 500 turns each. Drawing on experience with two–stage VTs [9,11,24,25], we set the rated voltage *U*_N_ = 1000 V, yielding a volts–per–turn value *e*_t_ = 1 V. The required cross–sectional area of core *C*_1_ was then calculated as:

(3) $$S=\frac{e_{t}\times 10^{4}}{4.44f_{S}B_{n}k_{S}}=47.4\;cm^{2}$$

where *f*_s_ is the operating frequency (50 Hz for this IVD) and *k*_s_ is the lamination stacking factor of the core (approximately 0.95). *B*_n_ is the rated magnetic flux density. For *C*_1_, *B*_n_ was set to 1.0 T to provide an adequate margin below the saturation flux density. Cold–rolled silicon steel, with a saturation flux density of up to 1.6 T, was selected for *C*_1_ because the first–stage core operates at the highest magnetic flux level and requires stable performance under relatively high–flux–density conditions. The magnetic characteristics of cold–rolled silicon steel is shown in Figure 5a.

Core *C*_2_, comprising four sections (upper, lower, inner, and outer), is positioned around *C*_1_ and *W*_1_. The gaps between adjacent sections are sealed with silicon steel tape. Serving as the second–stage core, *C*_2_ also provides effective magnetic shielding for *C*_1_; this arrangement is referred to as a closed shielded core structure, which significantly reduces the magnetic error.

The second–stage winding *W*_2_ is wound uniformly around core *C*_2_ using enameled copper wire. The parameters of the second–stage VT were calculated using the same procedure as that for the first stage and are therefore not repeated here. The excitation current of the second stage is much smaller than that of the first stage, and the magnetic flux density in *C*_2_ is correspondingly lower than that in *C*_1_. Under these low–flux–density conditions, high initial permeability is more important than high saturation flux density. Permalloy, with an initial relative permeability of approximately 10^5^, was therefore selected for *C*_2_ to reduce excitation current and enhance magnetic shielding. The magnetic characteristics of permalloy is shown in Figure 5b.

**Figure 6:**
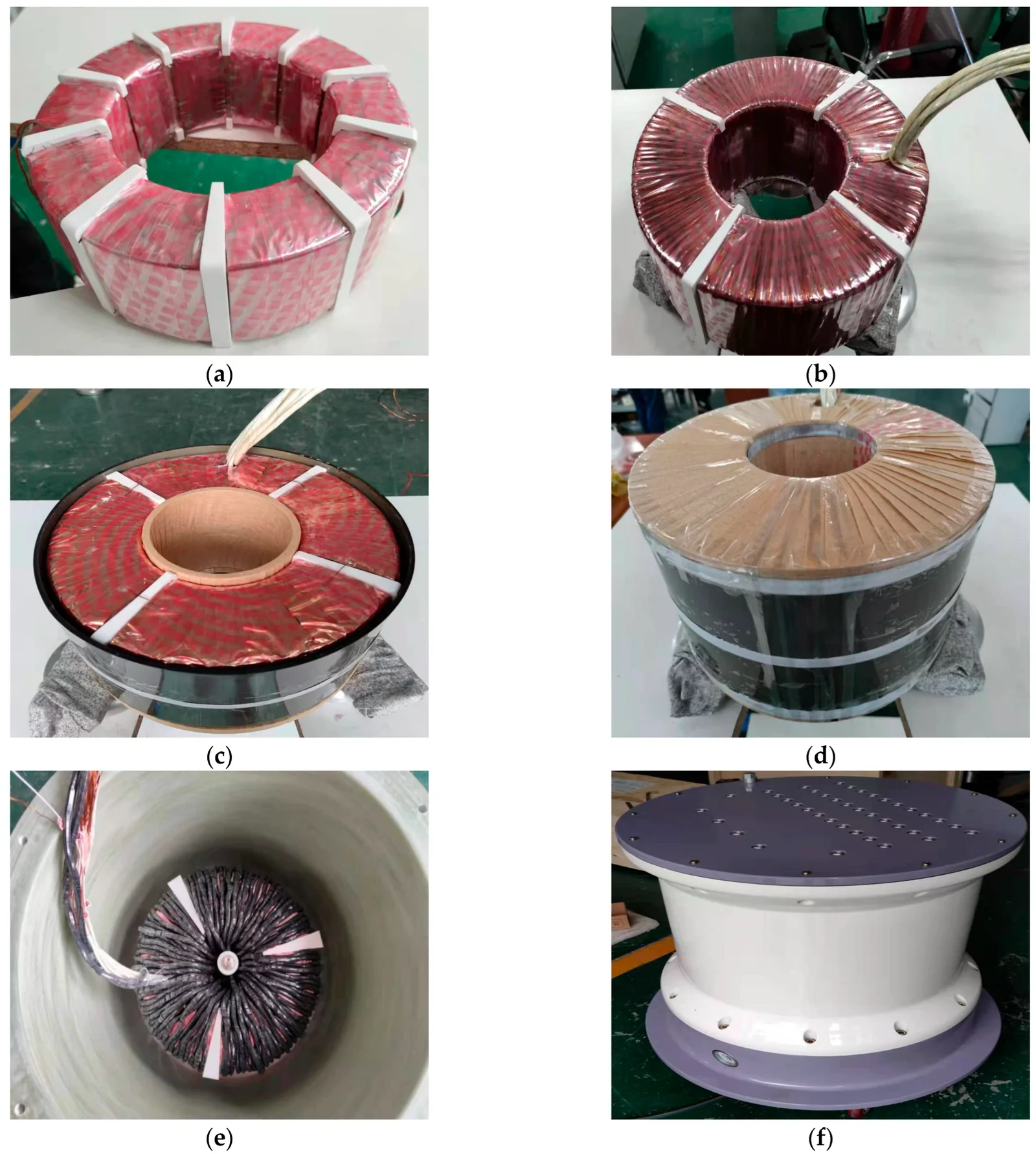
Photographs of the fabricated IVD: (**a**) first–stage VT; (**b**) second–stage VT; (**c**) segment of the third–stage VT core; (**d**) assembled third–stage VT core; (**e**) third–stage VT; (**f**) complete IVD.

The third–stage core *C*_3_ likewise comprises four sections and encloses both the second–stage core and winding. Since *C*_3_ also operates at a low magnetic flux density and mainly serves to further reduce excitation and magnetic coupling errors, it was fabricated from the same material as *C*_2_, namely permalloy. Winding *W*_3_ comprises 1100 turns of coaxial cable and is wound around the toroidal core *C*_3_ as 11 parallel strands of 100 turns each. After assembly of the three–stage cores and windings, the IVD was housed in a sealed insulating cylinder filled with dry nitrogen to reduce the influence of ambient humidity on the insulation and winding parameters. The IVD photographs of different stages of fabricating are shown in Figure 6.

## 4. Evaluation of Magnetic and Electrical Shielding

Capacitive and magnetic errors in the proposed IVD are suppressed by equipotential electrical shielding and a closed shielded core, respectively. Their effectiveness was evaluated experimentally by measuring ratio error and phase displacement under controlled shielding configurations.

The measurements were performed using an instrument transformer calibrator [28,29,30,31]. This device determines the complex ratio error between a test signal and a standard signal at the same nominal ratio; it does not measure the absolute voltage directly.

In the measurement process, the calibrator compares the amplitude and phase of the test signal *U*_X_ with those of the standard signal *U*_N_ by using *U*_ref_ as the reference channel. It then directly provides the ratio error *f* and phase displacement *δ*. In the present measurement setup, *U*_N_ and *U*_ref_ were derived from the same source; therefore, *U*_N_ = *U*_ref_, and the measured complex error can be expressed as follows:

(4) $$\varepsilon =\frac{U_{X}-U_{N}}{U_{\mathrm{ref}}}=f+j\delta$$

When using an instrument transformer calibrator, a standard signal with the same rated value as the signal under test is required. This is typically provided by a standard VT with the same rated ratio as the VT under test. The accuracy class of the standard VT must be at least two levels higher than that of the VT under test [32].

**Figure 7:**
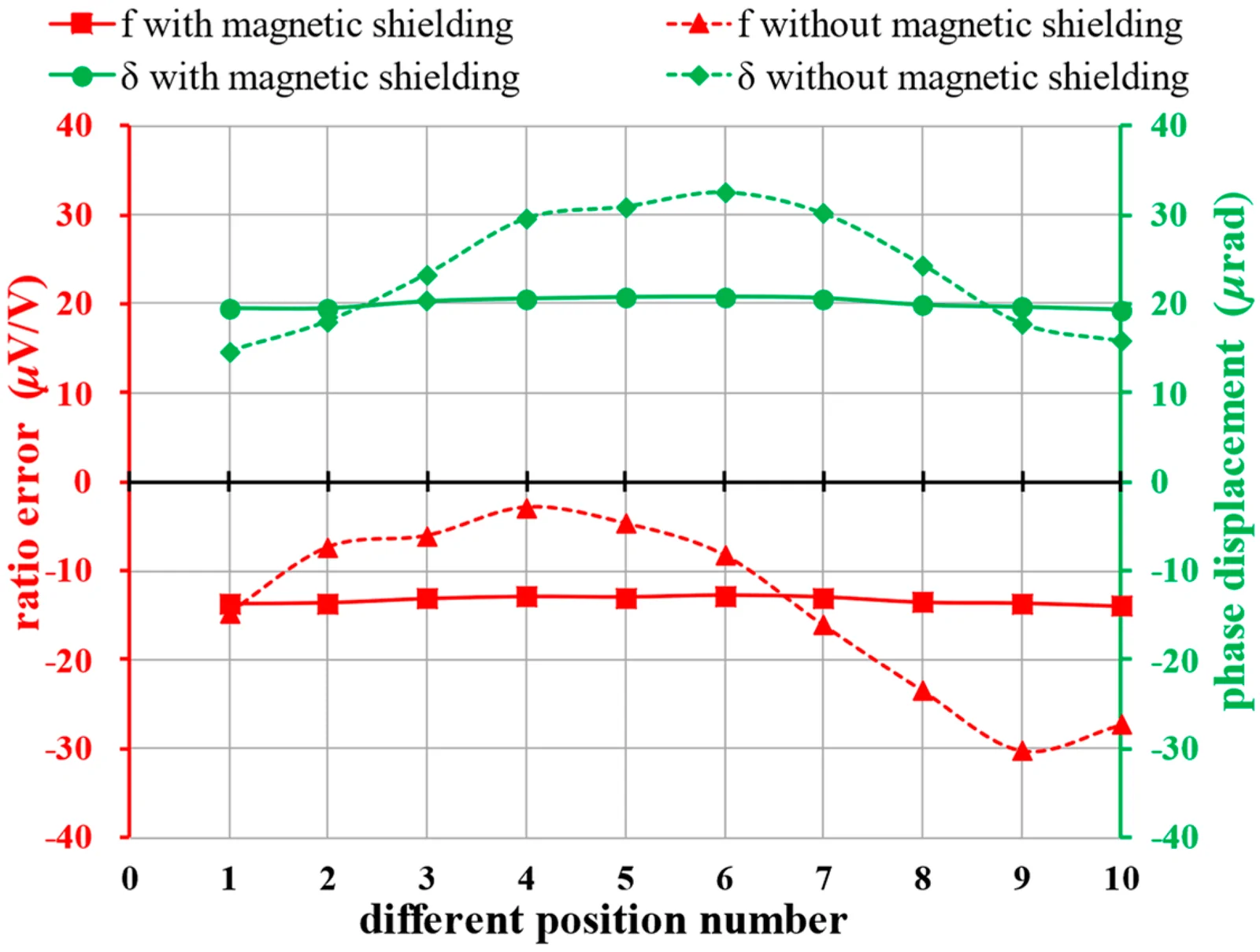
Effect of magnetic shielding provided by core *C*_2_ on the first–stage VT.

First, the magnetic shielding effectiveness of core *C*_2_ on the first–stage VT was evaluated. Because magnetic leakage is spatially nonuniform, the induced–voltage error depends on the angular position around the core. Ten measurement positions were uniformly distributed along the circumference of core *C*_1_ at angular intervals of approximately 36°. A single–turn sensing winding was placed at each position in sequence to measure the local induced–voltage error. Therefore, the horizontal axis in Figure 7 represents the position index from 1 to 10 along the circumference of core *C*_1_. The error distributions with and without core *C*_2_ shielding are shown in Figure 7.

Figure 7 shows that, in the absence of core *C*_2_, the error curves fluctuate significantly across different positions. The maximum spread in ratio error is approximately 30 μV/V, and that in phase displacement is approximately 30 μrad. With core *C*_2_ in place, the error curves flatten considerably, confirming that the magnetic shielding provided by *C*_2_ meets the design requirements.

**Figure 8:**
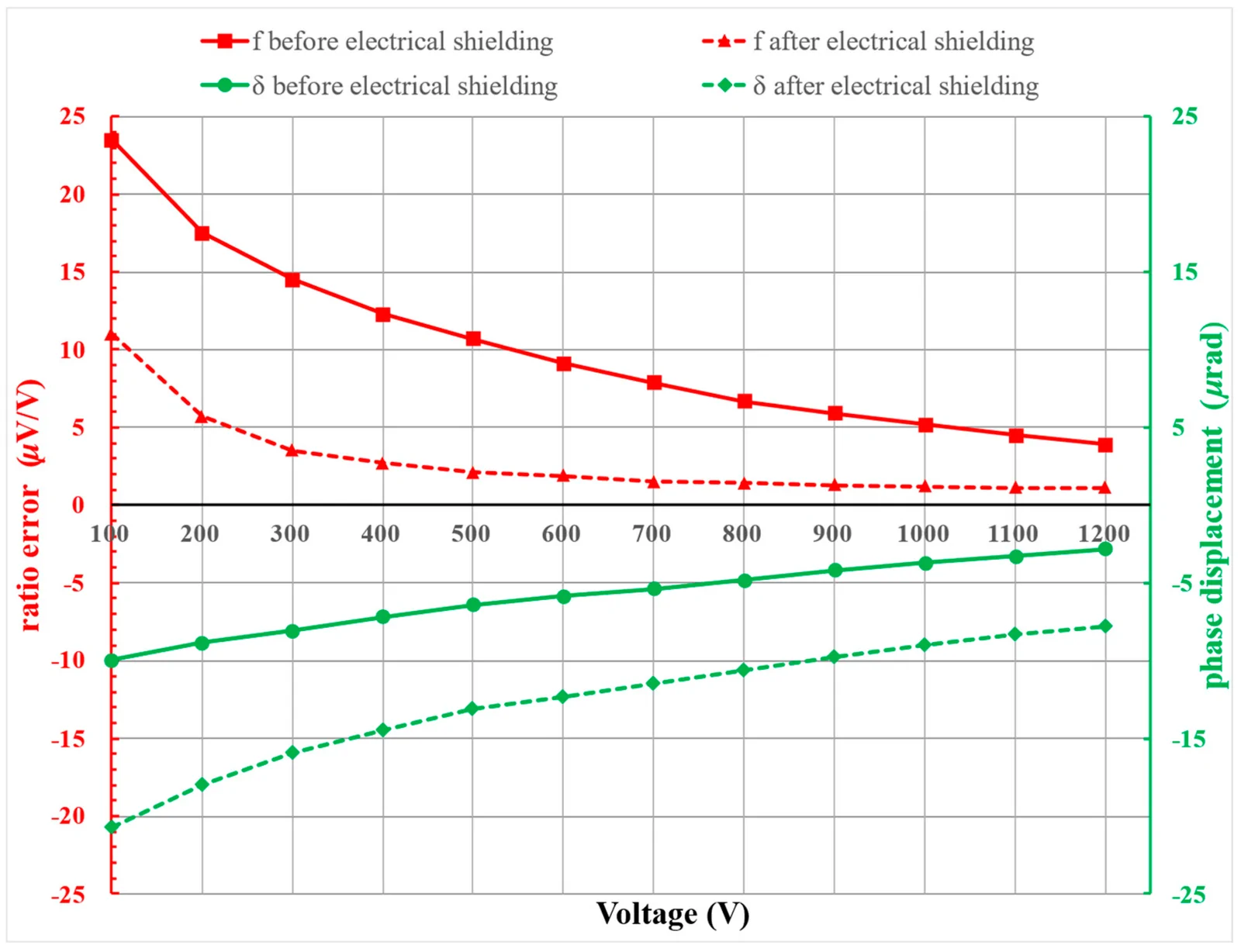
Effect of the copper–foil electrical shielding layer on the second–stage VT.

During fabrication, multiple electrical shielding layers of copper foil were incorporated, as shown in Figure 4. To verify the effectiveness of the electrical shielding, a 10–turn winding was uniformly wound around core *C*_2_ after completion of the second–stage VT. The error was measured at various voltages to compare results before and after adding the copper shielding. For this measurement, the magnetic shielding core *C*_3_ was not yet installed. The results are shown in Figure 8.

A comparison of errors before and after adding the second–stage electrical shielding clearly demonstrates that the shielding significantly reduces the errors. The dominant effect is observed in the phase displacement because electrical shielding mainly modifies the distributed capacitance, and its influence is therefore reflected primarily in the capacitive error component. The error of the two–stage VT is observed to lie in the fourth quadrant, characterized by a positive ratio error and a negative phase displacement. Based on two–stage VT theory, the total excitation error equals the negative product of the individual stage excitation errors:

(5) $$\begin{array}{ll} \varepsilon_{e2} & =-\frac{I_{02}Z_{02}}{U_{1}}\\ & =-\frac{I_{01}Z_{01}}{U_{1}}\frac{I_{02}Z_{02}}{I_{01}Z_{01}}\\ & =-\varepsilon_{1}\varepsilon_{2} \end{array}$$

**Figure 9:**
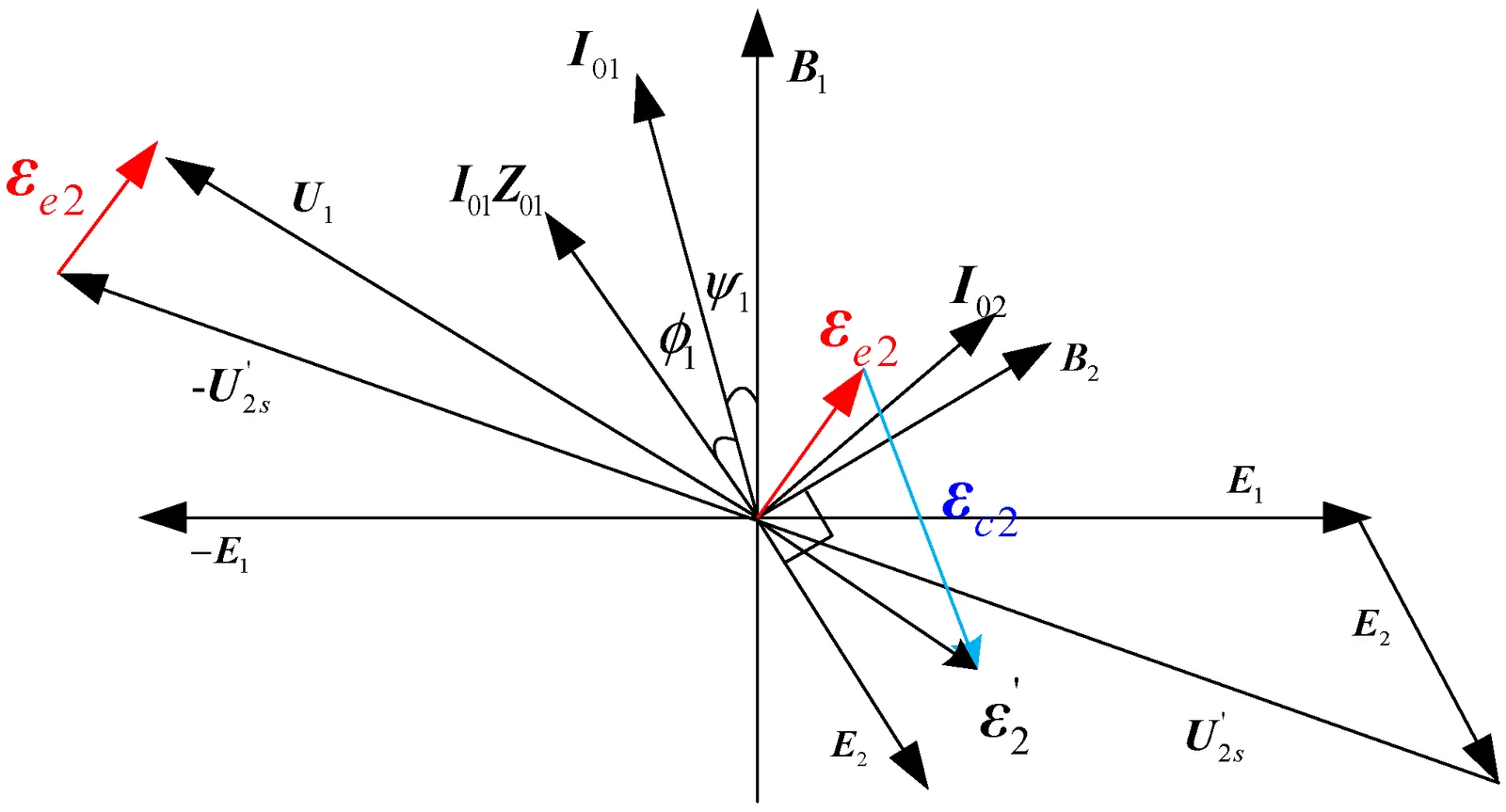
Phasor diagram of the two–stage VT.

The phasor diagram of the two–stage VT is shown in Figure 9, with *E*_1_ (the electromotive force of the first–stage VT) taken as the reference phasor. *B*_1_ represents the magnetic flux of core *C*_1_, which leads *E*_1_ by π/2. Consequently, the excitation current *I*_01_ and the voltage drop *I*_01_*Z*_01_ lead by angles *ψ*_1_ and *Φ*_1_, respectively, where *ψ*_1_ is attributable to core loss and *Φ*_1_ is the impedance angle of *Z*_01_. The phase relationship analysis for the second stage follows the same procedure as for the first stage.

The resulting $\varepsilon_{e2}$ represents the total excitation error of the two–stage VT. Theoretically, this error should reside in the first quadrant, with both the ratio error and phase displacement being positive. However, the ratio error and phase displacement of the first–stage VT are each less than 100 ppm. According to Equation (5), the two–stage VT error should be less than 0.01 ppm; yet the measured ratio error and phase displacement both exceeded 2 ppm. Considering both the phase and magnitude of the observed error, the fourth–quadrant error of the two–stage VT is attributed to the capacitive component $\varepsilon_{c2}$.

## 5. Integrated–Reference Bootstrap Self–Calibration

This section describes the integrated–reference bootstrap self–calibration method developed for the proposed 1 kV three–stage IVD. The calibration configuration and error equations are first presented. The expected influence of the compensation–free structure and integrated reference winding on long–term stability is then discussed, together with the limitations of the present study.

### 5.1. Integrated–Reference Bootstrap Self–Calibration Method

In conventional bootstrap self–calibration, the reference potential directly determines the calibration accuracy. A highly stable auxiliary transformer and additional guarding devices are therefore usually required to provide a stable reference and suppress shielding leakage [12,13,18]. Here, the reference winding is integrated into the ratio–winding assembly, allowing the reference potential to be generated within the divider itself.

**Figure 10:**
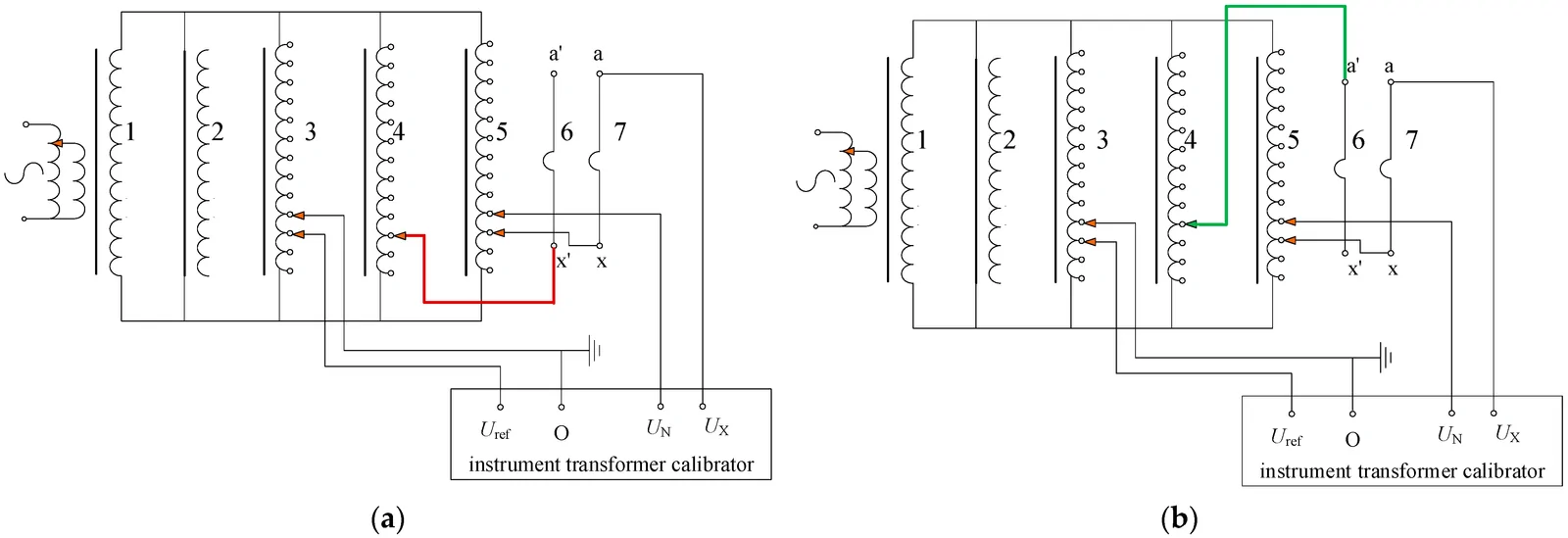
Schematic diagram of IVD self–calibration using the bootstrap method: (**a**) low–potential shielding; (**b**) high–potential shielding. 1: voltage booster, 2: first–stage winding, 3: second–stage winding, 4: shielding layer conductor of the third–stage coaxial–cable winding, 5: center conductor of the third–stage coaxial–cable winding, 6: shielding layer conductor of the reference winding formed by the 11th coaxial cable, 7: center conductor of the reference winding formed by the 11th coaxial cable.

As shown in Figure 10, the shielding winding and ratio winding correspond to the outer shield conductor and the center conductor of the coaxial cable of the third–stage VT, respectively. Both are composed of 10 sections connected in series after the parallel winding process.

The multi–wire parallel winding technique ensures that the electrical parameters of the 10 sections are nearly identical, thereby achieving a uniform and accurate potential distribution. Connecting the shielding winding and ratio winding in parallel to the power supply ensures equipotential conditions in the shielding layer, effectively eliminating capacitive errors due to leakage currents.

Let a′x′ and ax denote the outer shield conductor and center conductor of the reference winding (the 11th coaxial cable), respectively. This winding comprises 100 turns wound in parallel with the 10 coaxial cables of the third–stage VT, providing the reference potential. Since the shield potential of a′x′ can be set to either high–potential or low–potential shielding, error measurements under both configurations are required for each section of the ratio winding during self–calibration. The average of the two measurements is taken as the final error.

The self–calibration error calculation is described below, using the high–potential shielding configuration as an example. Let the reference potential be denoted as $E_{S}^{H}$ and its value relative to the rated value be $\varepsilon_{S}^{H}$.The actual error of each section potential is then given by:

(6) $$E_{i}^{H}=E_{S}^{H}(1+\varepsilon_{S}^{H})(1+\varepsilon_{i}^{H})\approx E_{S}^{H}(1+\varepsilon_{S}^{H}+\varepsilon_{i}^{H})$$

(7) $$\varepsilon_{Ai}^{H}=\varepsilon_{i}^{H}+\varepsilon_{S}^{H}$$

Summing the error equations for all sections yields:

(8) $$\sum_{i=1}^{N}\varepsilon_{Ai}^{H}=\sum_{i=1}^{N}\varepsilon_{i}^{H}+N\varepsilon_{S}^{H}$$

The left–hand side of Equation (8) represents the autotransformer winding error at a 1:1 ratio; thus:

(9) $$\sum_{i=1}^{N}\varepsilon_{Ai}^{H}=0$$

The error of the reference potential is:

(10) $$\varepsilon_{S}^{H}=-\frac{1}{N}\sum_{i=1}^{N}\varepsilon_{i}^{H}$$

The error of the *N*:*k* ratio under high–potential shielding is:

(11) $$\begin{array}{ll} \varepsilon_{N:k}^{H} & =\frac{1}{k}\sum_{i=1}^{k}\varepsilon_{i}^{H}+\varepsilon_{S}^{H}\\ & =\frac{1}{k}\sum_{i=1}^{k}\varepsilon_{i}^{H}-\frac{1}{N}\sum_{i=1}^{N}\varepsilon_{i}^{H} \end{array}$$

Similarly, under low–potential shielding, the error of the *N*:*k* ratio is:

(12) $$\begin{array}{ll} \varepsilon_{N:k}^{L} & =\frac{1}{k}\sum_{i=1}^{k}\varepsilon_{i}^{L}+\varepsilon_{S}^{L}\\ & =\frac{1}{k}\sum_{i=1}^{k}\varepsilon_{i}^{L}-\frac{1}{N}\sum_{i=1}^{N}\varepsilon_{i}^{L} \end{array}$$

The actual error of the *N*:*k* ratio is:

(13) $$\varepsilon_{N:k}=\frac{1}{2}(\varepsilon_{N:k}^{H}+\varepsilon_{N:k}^{L})$$

### 5.2. Discussion on Long–Term Stability

The compensation–free design is expected to reduce drift–sensitive elements in the ratio–reproduction path. Conventional compensation networks typically contain precision resistors, capacitors, or adjustable components whose temperature coefficients, dielectric absorption, humidity sensitivity, and aging behavior can introduce long–term changes in the voltage ratio. By avoiding these components, the proposed IVD reduces potential drift mechanisms associated with electronic compensation.

The integrated reference winding may further improve calibration robustness. Because the reference and ratio windings are made from the same type of coaxial cable, wound in the same process, and exposed to the same electromagnetic and environmental conditions, changes in conductor resistance, insulation properties, and distributed capacitance are expected to be correlated. During self–calibration, such correlated variations can appear partly as common–mode effects and may therefore be partially cancelled.

The sealed dry–nitrogen–filled enclosure also reduces humidity–related variations in insulation resistance and dielectric properties. However, no dedicated long–term drift test over several months has been performed in the present study. The stability benefit discussed here should therefore be interpreted as a design–based expectation rather than a quantitatively verified result. Long–term drift measurements under controlled environmental conditions will be conducted in future work.

## 6. Calibration and Uncertainty Evaluation

**Figure 11:**
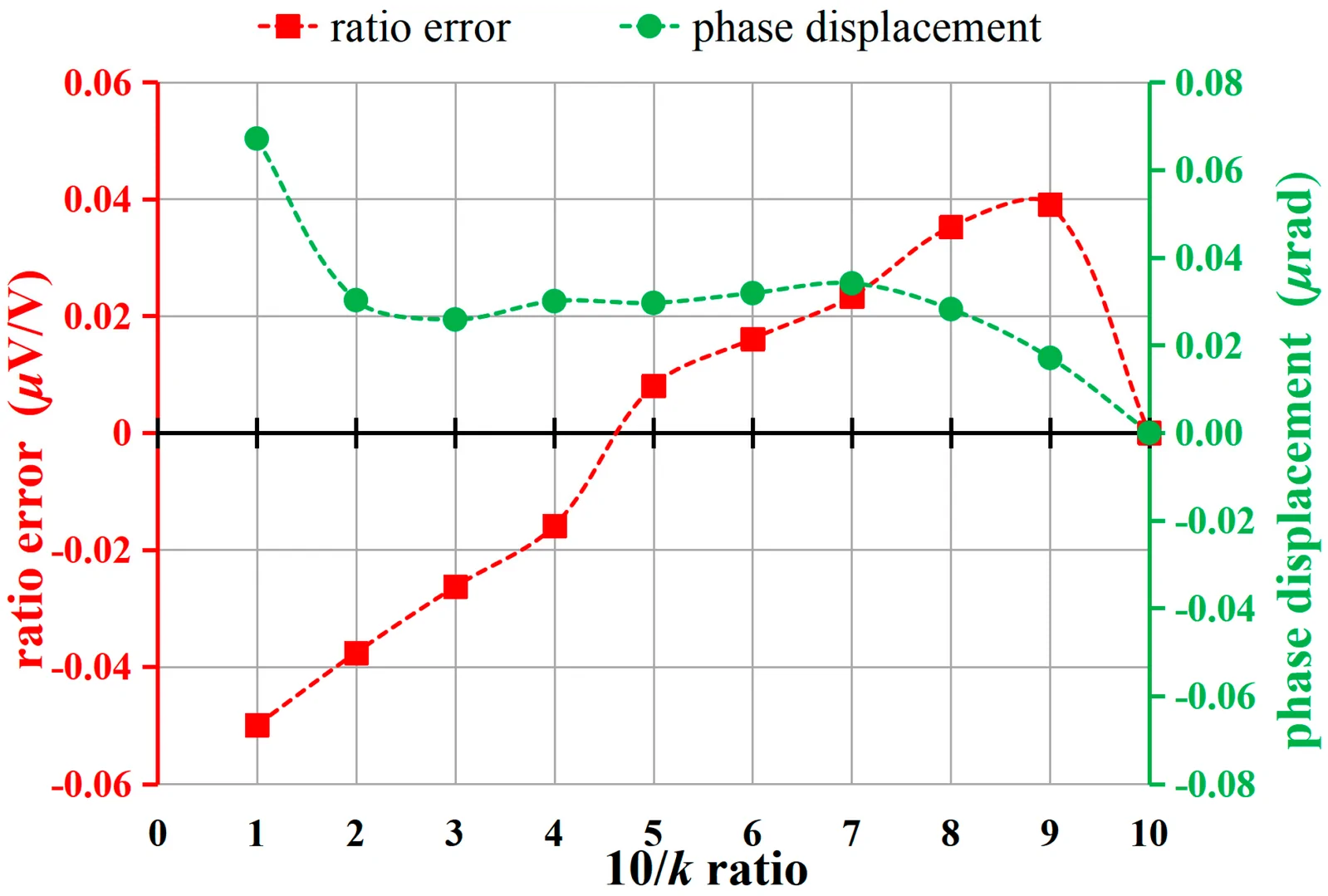
Calibration results of the IVD at 50 Hz.

The 1 kV IVD was calibrated at 50 Hz using the bootstrap method described above. The calibration results in Figure 11 show that, for ratios of 10:*k* (*k* = 1, 2, …, 10), the ratio error ranges from −0.050 μV/V to 0.040 μV/V and phase displacement ranges from 0.000 μrad to 0.072 μrad. Thus, the ratio error and phase displacement are within 0.08 μV/V and 0.08 μrad, respectively, across all taps.

The measurement uncertainty comprises four main components:

(1) Type A uncertainty (*n* = 12). The final result was obtained as the arithmetic mean of 12 monthly measurements taken over one year. From the standard deviation of these 12 measurements, the corresponding standard uncertainty components *u_f_*_1_ and *u_δ_*_1_ were determined to be 0.08 μV/V and 0.01 μrad, respectively.

(2) Measurement equipment. According to JJG–314 “Verification Regulation of Instrument Voltage Transformers” [32], the uncertainty contribution of the instrument transformer calibrator was evaluated. In this work, the calibrator was used as a comparison instrument for measuring the ratio error and phase displacement of the IVD, and its contribution to the uncertainty budget was classified as a Type B component. According to JJG–314, the contribution of the calibrator should not exceed 1/10 of the measurement error limit. Assuming a uniform distribution, the standard uncertainty contribution of the calibrator should be less than $0.1\varepsilon_{m\max}/\sqrt{3}$, where $\varepsilon_{m\max}$ denotes the corresponding measurement error limit. The corresponding standard uncertainty components introduced by the calibrator were denoted as *u_f_*_2_ and *u_δ_*_2_ for the ratio error and phase displacement, respectively, with values of 0.003 μV/V and 0.004 μrad.

(3) Environmental electromagnetic interference. Environmental electromagnetic interference was also considered as a Type B uncertainty component. According to JJG 314, the influence of electromagnetic interference from the power supply and surrounding equipment should be less than 1/10 of the error limit. Assuming a uniform distribution, the corresponding standard uncertainty components were denoted as *u_f_*_3_ and *u_δ_*_3_ for the ratio error and phase displacement, respectively. Their values were evaluated as 0.003 μV/V and 0.004 μrad.

(4) Shielding leakage. Although shielding leakage suppression measures were implemented during fabrication and calibration, the effect of residual shielding leakage on an IVD with 10^−7^–level accuracy cannot be neglected. The shielding leakage is therefore quantified experimentally.

A voltage is simultaneously applied to both ends of the primary side of the IVD. Under this condition, the primary–side potential difference is zero, and the output reflects the effect of shielding leakage.

The measured ratio error and phase displacement were 0.03 μV/V and 0.05 μrad. Assuming a uniform distribution, these values were divided by $\sqrt{3}$, yielding *u_f_*_4_ = 0.02 μV/V and *u_δ_*_4_ = 0.03 μrad.

The combined standard uncertainty is given by:

(14) $$u_{c}(f)=\sqrt{u_{f1}^{2}+u_{f2}^{2}+u_{f3}^{2}+u_{f4}^{2}}=0.02\;\mu V/V$$

(15) $$u_{c}(\delta )=\sqrt{u_{\delta 1}^{2}+u_{\delta 2}^{2}+u_{\delta 3}^{2}+u_{\delta 4}^{2}}=0.03\;\mu rad$$

The expanded uncertainty is calculated using the coverage factor *k*_c_ = 2:

(16) $$U_{f}=k_{c}u_{c}(f)=0.04\;\mu V/V\;\left(k_{c}=2\right)$$

(17) $$U_{\delta }=k_{c}u_{c}(\delta )=0.06\;\mu rad\left(k_{c}=2\right)$$

## 7. Wideband Error Results

**Figure 12:**
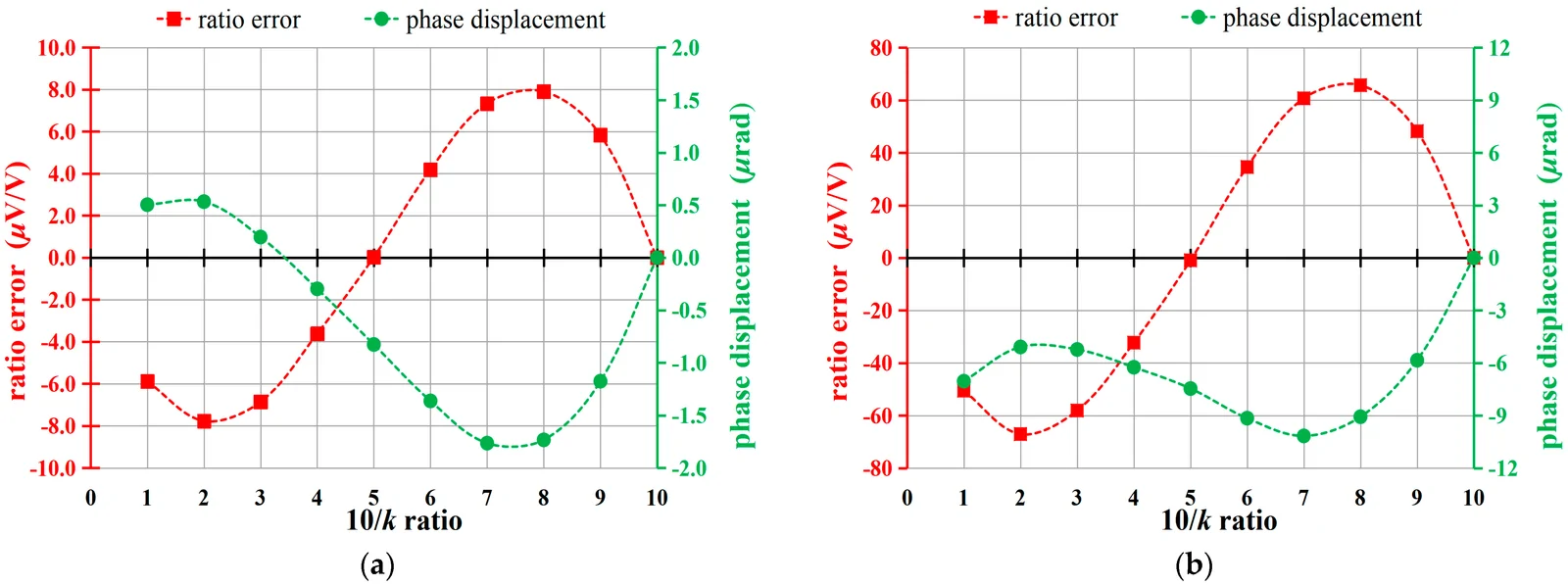
Wideband errors of the IVD: (**a**) 1 kHz; (**b**) 3 kHz.

The wideband error performance was evaluated at the rated input voltage of 1 kV for ratios of 10:*k* (*k* = 1, 2, …, 10), at frequencies up to 3 kHz. As shown in Figure 12, at 1 kHz, the ratio error ranges from −8 μV/V to 8 μV/V, and the phase displacement ranges from −9.2 μrad to 2.7 μrad. At 3 kHz, the ratio error increases to a range of −68 μV/V to 64 μV/V, while the phase displacement increases to a range of −68 μrad to −29 μrad.

Given the growing demand for accurate harmonic power measurements, harmonic voltage–ratio standards are increasingly required. The IVD developed in this study exhibits favorable wideband error characteristics, making it well suited to serve as a foundational voltage reference for harmonic voltage–ratio standards [33,34,35].

## 8. Conclusions

A 1 kV, 10–tap, compensation–free three–stage IVD was developed and experimentally evaluated. The divider combines multi–stage excitation, a closed shielded core, and equipotential coaxial–cable winding to suppress excitation, magnetic–coupling, and capacitive errors without using electronic compensation components. An integrated reference winding embedded in the third–stage coaxial–cable winding enables bootstrap self–calibration without a separate auxiliary IVD.

At 50 Hz, the calibrated ratio error and phase displacement are within 0.08 μV/V and 0.08 μrad, respectively, for all taps. The expanded uncertainties are 0.04 μV/V for ratio error and 0.06 μrad for phase displacement with *k*_c_ = 2. Wideband measurements show that, at 3 kHz, the ratio error ranges from −68μV/V to 64 μV/V and the phase displacement ranges from −68 μrad to −29 μrad, indicating the potential of the proposed IVD as a voltage–ratio reference for harmonic measurement and calibration.

The compensation–free structure is expected to reduce drift mechanisms associated with electronic compensation components. However, dedicated long–term drift tests have not yet been performed. Future work will therefore focus on long–term stability evaluation under controlled environmental conditions and on further optimization of the wideband error performance.

## Figures and Tables

**Table 1:** Comparison of representative IVDs and related voltage–ratio standards with the proposed IVD.

Reference	Voltage/Ratio	Frequency Range	Calibration Approach	Ratio Accuracy	Compensation
Zhou et al., 2014 [11]	1000 V; ratio/taps not fully stated	50 Hz–1000 Hz	Self–calibrating IVD; coaxial–cable winding	Ratio error better than 2 × 10^−7^	Frequency–division compensation; electronic compensation involved
Wang et al., 2018, 2022 [12,13]	1000 V; multi–decade IVD standard	Power frequency	Establishment/calibration of a 1000 V multi–decade IVD standard	Ratio error better than 5 × 10^−8^	Electronic compensation involved for classical 1 kV IVDs
Budovsky et al., 2004 [14]	1000 V; output ratios 0.001–0.01	40 Hz–1000 Hz; 50 Hz specifically in title	Calibration of 1000 V/50 Hz IVDs and ratio transformers	Wideband error within 1 × 10^−6^	Not explicitly stated, compensation/stability not the main focus
Small et al., 2005 [15]	1000 V; output ratios 0.001–0.01; precision three–stage IVD	40 Hz–1000 Hz; 50 Hz specifically in title	Calibration using 1000 V/50 Hz IVD/ratio–transformer calibration method	Wideband error within 1 × 10^−6^	Precision three–stage structure; electronic compensation not explicitly stated in the manuscript
Shao et al., 2024 [16]	10 kV IVD; ratio not stated	Power frequency	Self–calibration setup at power frequency	Not explicitly stated	Designed to bridge low–voltage standards and high–voltage transformers; compensation not explicitly stated
Yin et al., 2019 [17]	500/√3 kV three–stage VT; voltage ratio not stated	Power frequency	Traceability/calibration method for precision high–voltage VT	Not explicitly stated	Double–excitation structure; not described as electronic compensation in the manuscript
Beug and Moser, 2015 [18]	Two–stage coaxial IVD; voltage/ratio not stated	1 kHz–100 kHz	Dedicated calibration setup for high–frequency coaxial IVD	Not explicitly stated	Coaxial two–stage design to minimize capacitive errors; compensation mainly by structural/coaxial design
This work	1000 V; 10 taps; ratios of 10:k, k = 1~10	50 Hz–3 kHz	Integrated–reference bootstrap self–calibration method	≤0.08 μV/V and ≤0.08 μrad at 50 Hz; (−68–64) μV/V and (−68–−29) μrad at 3 kHz	Compensation–free three–stage structure; no electronic compensation components

Note: Only parameters explicitly reported in the cited literature are included. Parameters that are not clearly available from the cited sources are marked as “not explicitly stated”.

## Data Availability

The original contributions presented in this study are included in the article; further inquiries can be directed to the corresponding author.
